# Sensitive and Specific Immunohistochemistry Protocol for Nucleocapsid Protein from All Common SARS-CoV-2 Virus Strains in Formalin-Fixed, Paraffin Embedded Tissues

**DOI:** 10.3390/mps4030047

**Published:** 2021-07-10

**Authors:** Yunguang Sun, Linna Ge, Sameer S. Udhane, John F. Langenheim, Mary J. Rau, Mollie D. Patton, Alexander J. Gallan, Juan C. Felix, Hallgeir Rui

**Affiliations:** Department of Pathology, Medical College of Wisconsin, TBRC-C4980, 8701 Watertown Plank Rd, Milwaukee, WI 53226, USA; ysun@mcw.edu (Y.S.); lge@mcw.edu (L.G.); sudhane@mcw.edu (S.S.U.); jlangenheim@mcw.edu (J.F.L.); mrau@mcw.edu (M.J.R.); mpatton@mcw.edu (M.D.P.); agallan@mcw.edu (A.J.G.); jcfelix@mcw.edu (J.C.F.)

**Keywords:** SARS-CoV-2, immunohistochemistry protocols, nucleocapsid protein, spike protein

## Abstract

Human coronavirus disease 2019 (COVID-19) is a life-threatening and highly contagious disease caused by coronavirus SARS-CoV-2. Sensitive and specific detection of SARS-CoV-2 viral proteins in tissues and cells of COVID-19 patients will support investigations of the biologic behavior and tissue and cell tropism of this virus. We identified commercially available affinity-purified polyclonal antibodies raised against nucleocapsid and spike proteins of SARS-CoV-2 that provide sensitive and specific detection of the virus by immunohistochemistry in formalin-fixed, paraffin-embedded tissue. Two immunohistochemistry protocols are presented that are mutually validated by the matched detection patterns of the two distinct viral antigens in virus-infected cells within autopsy lung tissue of COVID-19 deceased patients. Levels of nucleocapsid protein in the lungs of COVID-19 decedents, as measured by quantitative histo-cytometry of immunohistochemistry images, showed an excellent log–linear relationship with levels of viral nucleocapsid RNA levels, as measured by qRT-PCR. Importantly, since the nucleocapsid protein sequence is conserved across all known viral strains, the nucleocapsid immunohistochemistry protocol is expected to recognize all common variants of SARS-CoV-2. Negative controls include autopsy lung tissues from patients who died from non-COVID-19 respiratory disease and control rabbit immunoglobulin. Sensitive detection of SARS-CoV-2 in human tissues will provide insights into viral tissue and cell distribution and load in patients with active infection, as well as provide insight into the clearance rate of virus in later COVID-19 disease stages. The protocols are also expected to be readily transferable to detect SARS-CoV-2 proteins in tissues of experimental animal models or animals suspected to serve as viral reservoirs.

## 1. Introduction

In December of 2019, an outbreak of pneumonia cases of unknown etiology occurred in Wuhan, China, leading to the identification of a novel beta-coronavirus, named SARS-CoV-2, as the causative agent [1,2,3]. The infectious disease was termed COVID-19 in January of 2020 by the World Health Organization [4]. COVID-19 patients with lower respiratory tract infection often develop acute respiratory disease syndrome (ARDS) that is also observed in patients with severe acute respiratory syndrome (SARS) and Middle East respiratory syndrome (MERS), other corona-viral pneumonias. However, COVID-19 is much more transmissible between people than SARS and MERS, and was declared a pandemic in February of 2020. While great progress has been made to develop effective vaccines, worldwide vaccination efforts will take years, and treatment options remain limited.

The SARS-CoV-2 virus causes a wide spectrum of clinical manifestations in patients with COVID-19 [5]. For rational development of treatments for COVID-19 there is a great need to understand the pathogenesis and pathology of COVID-19, not only in the respiratory tract, but also in numerous other organs that become directly infected through vascular or neuronal spread. Commonly affected non-respiratory organs include GI-tract, kidney, heart, skin, and central nervous system [6,7,8]. In addition, numerous pathological changes appear secondary to, or indirectly from, viral infection, including aberrant immune cell activation, vascular changes, and coagulopathies, in turn affecting numerous organs [9,10,11].

Sensitive tools are needed to determine the virus distribution in organs and cells [12,13], and to identify associated adaptations in the proximal microenvironment, including immune cell recruitment and local inflammatory changes. Immunohistochemistry for viral protein provides a rapid means to identify virally infected cells within histological sections of formalin-fixed, paraffin-embedded (FFPE) tissues. Initial IHC assays with the ability to detect SARS-CoV-2 were based on antibodies originally raised against SARS-CoV proteins with sufficient cross-reactivity to SARS-CoV-2 epitopes [14,15]. While there is general agreement that virus presence in the lungs is critical for initiating lung damage, and that lung damage continues to progress even after the virus is cleared [16,17], controversies continue with regard to the transience or duration of active viral infection and its effect on disease progression. One autopsy study reported IHC-detectable viral spike protein in lungs of 57% of COVID-19 fatalities (13/23) and concluded that “viral infection in areas of ongoing active injury contributes to persistent and temporally heterogeneous lung damage” [18], while another autopsy study used nucleocapsid protein immunohistochemistry and detected virus in only 7% (1/14) of COVID-19 decedents, and concluded that “direct viral tissue damage is a transient phenomenon that is generally not sustained throughout disease progression” [19].

## 2. Experimental Design

Formalin-fixed, paraffin-embedded (FFPE) samples of lungs collected during autopsy from COVID-19 deceased patients or non-COVID-19 deceased patients were made available through the Medical College of Wisconsin Tissue Bank under IRB approved protocol. The main experimental stages included generation of histology sections, antigen retrieval, incubation with multiple affinity-purified polyclonal rabbit antibodies directed to SARS-CoV-2 proteins, kindly provided by ProSci (ProSci, Poway, CA, USA), over a range of serial dilutions, followed by standard visualization by DAB-chromogen deposition via horse radish peroxidase (HRP)-linked secondary reagents. For protocol development, we used a DAKO/Agilent Omnis autostainer, but the steps are directly adaptable to manual immunohistochemistry staining or staining using alternative robots. Digital images of stained slides were obtained on a 3DHistech Pannoramic scanner, but standard brightfield microscopes or other scanners can be used.

**Figure 1:**
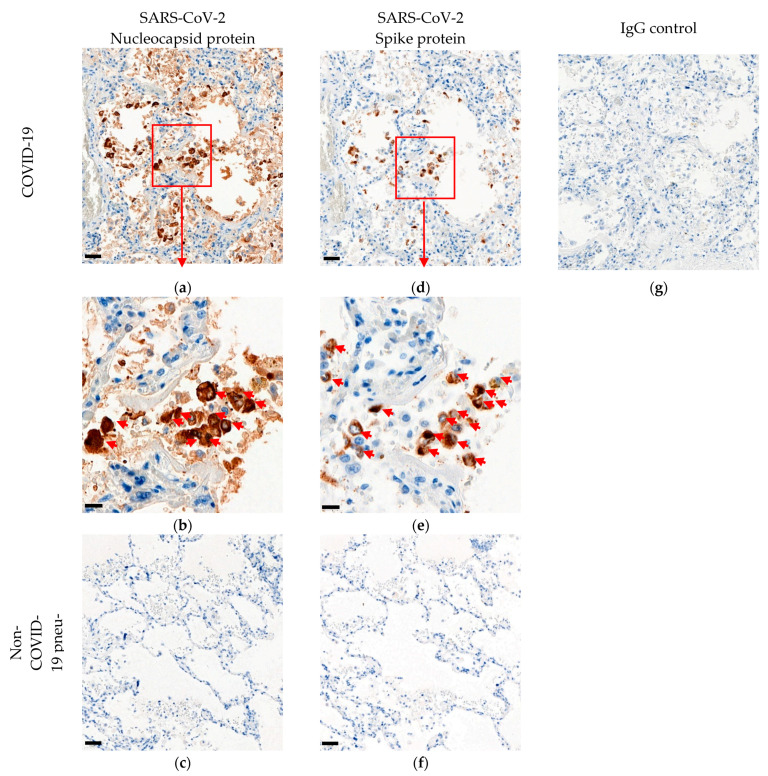
Immunohistochemistry (IHC) of SARS-CoV-2 antigens in FFPE lung tissue. Detection of SARS-CoV-2 nucleocapsid protein ((**a**,**b**); brown staining, red arrows) or SARS-CoV-2 spike protein ((**d**,**e**); brown staining, red arrows) in adjacent sections of autopsy lung tissue from COVID-19 deceased patient. Negative control staining on autopsy lung tissue from patient who died from non-COVID-19 pneumonia is shown for nucleocapsid protein (**c**) or spike protein (**f**). Negative control using normal rabbit immunoglobulin on COVID-19 autopsy tissue is presented (**g**). DAB chromogen and hematoxylin counterstain are used. Scale bars: 50 µM in (**a**,**c**,**d**,**f**,**g**); 20 µM in (**b**,**e**).

**Figure 2:**
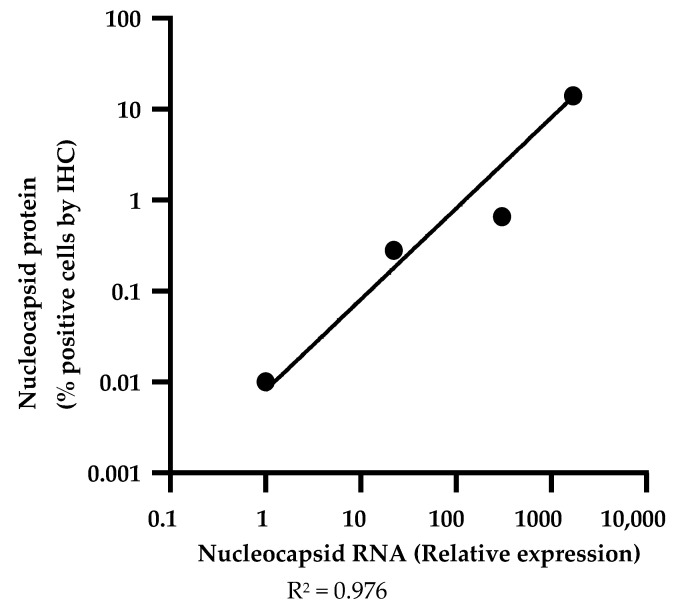
Levels of nucleocapsid protein by immunohistochemistry correlate well with transcript levels in COVID-19 lung tissues. Log-linear relationship is observed between proportion of nucleocapsid-positive cells by immunohistochemistry of histological sections of FFPE lung tissue and relative *nucleocapsid* RNA levels in adjacent sections of lung samples from four COVID-19 decedents. IHC image analysis and quantitation was performed using QuPath and correlation of IHC and mRNA expression was analyzed using Prism software. There was no evidence of post-mortal degradation of the nucleocapsid protein antigen or RNA, as assessed by time from death to autopsy in the four cases (listed in order of decreasing nucleocapsid protein/RNA: A50-47.5 h; B66-25.2 h; C80-34.1 h, D60-9.12 h).

Since there was no positive control tissue available with known expression of viral proteins, we used a strategy of reciprocal cross-validation of the immunohistochemistry protocols by documenting corresponding staining patterns of the antibody directed to the nucleocapsid protein and the antibody directed to the spike protein in lungs from patients who died with known acute phase COVID-19 (Figure 1). Rabbit immunoglobulin served as negative control for the primary antibodies, whereas FFPE sections of lungs from autopsies of known non-COVID-19 pneumonia decedents served as negative control tissues. We further validated the nucleocapsid protein immunohistochemistry protocol by documenting that levels of nucleocapsid protein in lungs of COVID-19 decedents, as measured by quantitative histo-cytometry of immunohistochemistry images (QuPath software, Ver 0.2.3), showed an excellent log–linear relationship with levels of the corresponding viral RNA, as measured by qRT-PCR of RNA isolated from adjacent tissue sections for four cases with variable expression by IHC, and a non-COVID-19 autopsy case was used as negative control (Figure 2).

qRT-PCR for nucleocapsid RNA was performed as follows: RNA was isolated from COVID-19 patients’ FFPE lung samples. Microtome sections totaling ≤80 μm tissue thickness were used for the RNA extraction. RNA was isolated using the RecoverAll^TM^ total nucleic acid isolation kit for FFPE (Thermo Fisher Scientific, Waltham, MA, USA) according to the manufacturer’s instructions. A total of 0.5 μg of RNA was reverse-transcribed to cDNA using SuperScript^TM^ III First-Strand Synthesis System (Thermo Fisher Scientific). qRT-PCR analysis was performed on the Bio-Rad CFX96 Dx Real-Time PCR detection system (BIO-RAD, Hercules, CA, USA) using 2019-nCov primer kit RUO (Integrated DNA Technologies (IDT), Coralville, IA, USA). Primers used to detect SARS-CoV-2 virus nucleocapsid mRNA were forward primer 5′-GAC CCC AAA ATC AGC GAA AT-3′, reverse primer 5′-TCT GGT TAC TGC CAG TTG AAT CTG-3′, and probe FAM-ACC CCG CAT/ZEN/TAC GTT TGG TGG ACC-3IABkFQ. Briefly, qRT-PCR was performed in 96-well plates using 50 ng/well cDNA and master mix of 1.5 μL of IDT probe/primer mix (IDT) and PCR supermix (Thermo Fisher Scientific) in a total volume of 20 μL. Amplification curves and the mean cycle threshold (Ct) values were calculated using the CFX Manager Dx software (BIO-RAD), and correction for the endogenous gene, ΔCt and ΔΔCt, were calculated. Fold change in gene expression for a particular gene was calculated by the 2^−ΔΔCt^ method [20].

Once we had identified SARS-CoV-2 positive autopsy tissues, we generated mini-tissue arrays that included lung tissue from both a SARS-CoV-2 protein-positive autopsy and negative control tissue from a non-COVID-19 pneumonia autopsy. Slides with positive and negative controls can be provided upon request to users of the SARS-CoV-2 immunohistochemistry protocols described here, as well as other SARS-CoV-2 protocols.

### 2.1. Materials

- Microscope Slides (Superfrost Plus microscope slides, Fisher Scientific, Pittsburgh, PA, USA; Cat. No.12-550-15);
- Paraffin-embedded tissue blocks;
- Clearify^TM^ (Agilent, Santa Clara, CA, USA; Cat. No. GC81030-2);
- EnVision^TM^ FLEX Target Retrieval Solution Low pH (50×; Agilent; Cat. No. GV80511-2);
- EnVision^TM^ FLEX Mini Kit (Agilent; Cat. No. GV82311-2). Components: DAB+ Chromogen Solution; DAB Substrate Buffer; FLEX HRP (polymer); Peroxidase Blocking Solution; Target Retrieval Solution, High pH, Concentrated 50×;

**Note:** Target Retrieval Solution, High pH, Concentrated 50× is not used, use instead: Target Retrieval Solution, Low pH, 50× (Agilent; Cat. No. GV80511-2);

- Protein Block (BioGenex, Fremont, CA, USA; Cat. No. HK112-9K);
- Hematoxylin (Agilent; Cat. No. GC80811-2);
- Antibody Diluent (Agilent; Cat. No. S080983-2);
- Antibodies:
  (a) Anti-SARS-CoV-2 nucleocapsid protein antibody (affinity-purified rabbit IgG; ProSci, Poway, CA, USA; Cat. No. 9099) diluted to a final concentration of 0.02 µg/mL.
  (b) Anti-SARS-CoV-2 spike S1 glycoprotein antibody (affinity-purified rabbit IgG (ProSci; Cat. No. 9083,) diluted to a final concentration of 1.0 µg/mL.
  (c) Rabbit immunoglobulin control (Vector Labs, Burlingame, CA, USA; Cat. No. I-1000-5), diluted to a final concentration of 1.0 or 0.02 µg/mL, depending on protocol.

### 2.2. Equipment

- Dako Omnis autostainer (Agilent, Santa Clara, CA, USA);
- Panoramic 250 slide scanner (3DHistech, Budapest, Hungary).

## 3. Procedure

Microtome sectioning. Time for completion: 5 min of sectioning time per FFPE tissue block, plus 60 min of slide baking.

1. Section paraffin-embedded tissue blocks of samples of interest and positive and negative SARS-CoV-2 control tissues by a standard microtome into 4 μm sections.
2. Mount sections on poly-L-lysine-charged glass slides and bake for 60 min at 62 °C.

Immunostaining. Time for completion: 2.5 h, depending on how many slides are stained. **Note:** Steps 3–10 are described as performed on the robotic Omnis autostainer with built-in antigen retrieval chamber (Dako/Agilent). Modify as needed for other systems.

3. Dewax slides with Clearify^TM^ clearing agent at 25 °C for 10 s.
  3.1. Wash.
4. Perform target retrieval using EnVision^TM^ FLEX Target Retrieval Solution, Low pH (6.0), 97 °C for 30 min.
  4.1. Wash.
5. Incubate with Protein Block for 30 min at room temperature (RT).
  5.1. Wash.
6. Dilute the primary antibodies or control antibodies with Antibody Diluent, incubate slides for 30 min at RT, and wash.
  6.1. Dilute anti-SARS-CoV-2 nucleocapsid protein antibody (affinity-purified rabbit IgG; ProSci; Cat. No. 9099) to a final concentration of 0.02 µg/mL.
    6.1.1. Dilute rabbit immunoglobulin control to a final concentration of 0.02 µg/mL (Vector Labs, Burlingame, CA, USA).
  6.2. OPTIONAL STEP. Dilute anti-SARS-CoV-2 spike S1 glycoprotein antibody (affinity-purified rabbit IgG (ProSci; Cat. No. 9083) to a final concentration of 1 µg/mL.
    6.2.1. OPTIONAL STEP. Dilute rabbit immunoglobulin control to a final concentration of 1 µg/mL (Vector Labs, Burlingame, CA, USA).
7. Perform endogenous peroxidase block with EnVision^TM^ FLEX Peroxidase Blocking Reagent for 3 min.
  7.1. Wash.
8. Add secondary reagent, EnVision^TM^ FLEX/HRP, for 30 min.
  8.1. Wash.
9. Add chromogen substrate, EnVision^TM^ FLEX Substrate Working Solution, for 5 min.
  **Note:** Brown coloration of tissues and cells represents positive staining.
  9.1. Wash.
10. Counterstain with Hematoxylin for 6 min.
  10.1. Wash.
  Post-staining treatment and image capture. Time for completion: 60 min.
11. Dehydrate sections through an ethanol series to xylene and coverslip slides.
12. Capture images of stained slides by 3DHistech Pannoramic scanner (Figure 1), other slide scanner, or brightfield microscope.

## 4. Expected Results

The described immunohistochemistry protocols for SARS-CoV-2 nucleocapsid protein and spike protein are both expected to provide sensitive detection of SARS-CoV-2 infected cells in FFPE tissues from patients or animals with active infection. As the virus is cleared by the immune system over days and weeks, tissues that were previously positive will become negative. We are working on a larger cohort of tissues to determine how fast the viral proteins and ribonucleic acids are cleared from lungs and other tissues. The most highly positive tissues are from early, acute phase of infection, and it is critical to include positive and negative control tissues to make confident conclusions. In lungs of COVID-19 decedents who died during the early, active viral infection period, we detected virus protein primarily in pneumocytes, with lower levels detectable in macrophages and within hyaline membranes of collapsing alveoli.

We further expect that the nucleocapsid immunohistochemistry protocol will detect all SARS-CoV-2 variants ((B.1.617.1/2 (India), B.1.351 (South Africa), B.1.1.7/B.1.351/P.1 (Japan/Brazil), B.1.616 (France), B.1.427/9 (United States–California), and B.1.1.7 (United Kingdom)), since the antibody recognizes an epitope within a highly conserved amino acid sequence K236-T280 of the nucleocapsid protein (Morey Setareh, ProSci Inc, personal communication). The antibody to spike protein recognizes an epitope within the amino acid sequence T19-S50, and ELISA data shows that it fails to recognize the P.1 (Brazil) variant (Morey Setareh, ProSci Inc, personal communication) due to its T20N/P26S mutations. In addition, *spike* subgenomic RNA (sgRNA) and protein are three times less abundant than the *nucleocapsid* sgRNA and protein [21,22]. Therefore, for reasons of broad SARS-CoV-2 variant coverage, the double validation of the nucleocapsid protein against both nucleocapsid RNA and spike protein, the higher nucleocapsid immunohistochemistry assay sensitivity, and the greater abundance of the nucleocapsid protein than the spike protein, we recommend the nucleocapsid protein immunohistochemistry protocol over the spike protein assay for sensitive and robust detection of SARS-CoV-2 virus in tissues.

Future work will explore viral protein levels in other tissues than lungs, including kidney, heart, and brain. While the present IHC protocols for SARS-CoV-2 proteins were developed for human tissues, we expect that the protocols will be directly transferable to other species, including experimental rodent or primate models, or species that may serve as viral reservoirs, including mink, felines, or bats.

## Data Availability

Original images and raw data are available.
